# Effects of acute hypoxia on human adipose tissue lipoprotein lipase activity and lipolysis

**DOI:** 10.1186/s12967-016-0965-y

**Published:** 2016-07-15

**Authors:** Bimit Mahat, Étienne Chassé, Jean-François Mauger, Pascal Imbeault

**Affiliations:** Behavioral and Metabolic Research Unit, School of Human Kinetics, Faculty of Health, Sciences, University of Ottawa, 125, University Street (room 350), Ottawa, ON K1N 6N5 Canada

**Keywords:** Intermittent hypoxia, Obstructive sleep apnea, Adipose tissue metabolism, Postprandial lipemia, Cardiovascular disease

## Abstract

**Background:**

Adipose tissue regulates postprandial lipid metabolism by storing dietary fat through lipoprotein lipase-mediated hydrolysis of exogenous triglycerides, and by inhibiting delivery of endogenous non-esterified fatty acid to nonadipose tissues. Animal studies show that acute hypoxia, a model of obstructive sleep apnea, reduces adipose tissue lipoprotein lipase activity and increases non-esterified fatty acid release, adversely affecting postprandial lipemia. These observations remain to be tested in humans.

**Methods:**

We used differentiated human preadipocytes exposed to acute hypoxia as well as adipose tissue biopsies obtained from 10 healthy men exposed for 6 h to either normoxia or intermittent hypoxia following an isocaloric high-fat meal.

**Results:**

In differentiated preadipocytes, acute hypoxia induced a 6-fold reduction in lipoprotein lipase activity. In humans, the rise in postprandial triglyceride levels did not differ between normoxia and intermittent hypoxia. Non-esterified fatty acid levels were higher during intermittent hypoxia session. Intermittent hypoxia did not affect subcutaneous abdominal adipose tissue lipoprotein lipase activity. No differences were observed in lipolytic responses of isolated subcutaneous abdominal adipocytes between normoxia and intermittent hypoxia sessions.

**Conclusions:**

Acute hypoxia strongly inhibits lipoprotein lipase activity in differentiated human preadipocytes. Acute intermittent hypoxia increases circulating plasma non-esterified fatty acid in young healthy men, but does not seem to affect postprandial triglyceride levels, nor subcutaneous abdominal adipose tissue lipoprotein lipase activity and adipocyte lipolysis.

## Background

Obstructive sleep apnea (OSA) is a prevalent sleep disorder affecting approximately 5–15 % of middle-aged and older adults in the general population [1]. Individuals with OSA experience short periods of hypopnea, inducing intermittent hypoxia-hypercapnia/normoxia cycles. The most salient symptom of OSA is excessive daytime sleepiness, but its most important health consequence is an approximate two-fold increased risk of developing cardiovascular disease (CVD) such as coronary artery disease, heart failure, or stroke [2]. The link between OSA and CVD could be explained by the fact that OSA may disturb lipid metabolism and lead to a deteriorated blood lipid profile. It has been shown that individuals with OSA display increased triglyceridemia (by ~30 %), independent of age and body mass index, compared to individuals without OSA [3].

Adipose tissue plays a central role in energy substrate homeostasis by acting as a crucial regulator of whole-body lipid flux. More specifically, in response to metabolic demand, triglyceride (TG) stored within adipocytes can be hydrolyzed into fatty acids and glycerol to be released for use by non-adipose organs. Postprandially, the transport of lipoprotein lipase (LPL) from intracellular vacuoles to the capillaries endothelium promotes the hydrolysis of dietary TG and subsequent uptake of dietary fatty acids within adipocytes [4, 5]. The proper regulation of lipid uptake and secretion by the adipose tissue is thought to be critical to limit ectopic fat storage in metabolically important tissues, namely the liver, skeletal muscles, and pancreatic beta cells, and to prevent chronic disorders such as type 2 diabetes and CVD [6, 7].

Recent animal studies demonstrated that chronic intermittent [8, 9] and acute hypoxia [10] increase hepatic TG secretion in the fasted state and delay TG clearance in the postprandial state. These changes appear to be caused, in part, by (a) an increase in lipid influx to the liver due to an increase in adipose tissue lipolysis and by (b) a suppression of LPL activity by more than 50 %. While the increase in adipose tissue lipolysis has been linked to the increase in sympathetic drive observed during hypoxia, the reduction in adipose tissue LPL activity appears to be explained by the upregulation of an important post-translational repressor of LPL, angiopoietin-like protein 4 (ANGPTL4) [9].

Despite evidence from animal studies indicating that hypoxia considerably affects adipose tissue functions, blood lipid profile, and potentially the risk of CVD or type 2 diabetes in OSA patients, data regarding these effects in humans is crucially lacking. Therefore, the objective of this study was to investigate the effects of hypoxia on human adipose tissue LPL activity and adipocyte lipolysis. We hypothesize that: (1) In differentiated human preadipocytes, acute exposure to hypoxia inhibits LPL activity, and (2) In humans, acute intermittent hypoxia leads to an exaggerated elevation in postprandial TG concentrations consequent to an increase in adipocyte lipolysis and/or an impairment in subcutaneous abdominal adipose tissue LPL activity.

## Methods

### In vitro experiments

#### Culture of human preadipocytes

Cryopreserved subcutaneous abdominal preadipocytes from two Caucasian female (average age: 39 y; mean body mass index: 22.74 kg/m^2^) were obtained from Zen-bio (NC, USA) and differentiated according to manufacturer’s instructions [11]. Briefly, preadipocytes were plated at a density of 4 × 10^4^ cells/cm^2^ in 24-well plates, and proliferated in preadipocytes medium (PM-1) for 48 h, or until confluence was reached. Differentiation was induced by substituting the culture media for adipocyte differentiation medium (DM-2) in which cells were maintained for 7 days. Cells were then fed by replacing the culture medium with the adipocyte maintenance medium (AM-1), and maturation was continued for another week. Fourteen days post-induction, cells were transferred to basal medium (BM-1) and incubated in either hypoxic (3 % oxygen) or normoxic (21 % oxygen) conditions [12], for 24 h. No cell lost was observed at the end of each treatment. After treatments, media were collected and cells were washed three times with phosphate buffer saline (PBS). To assess LPL activity, cells were incubated for 30 min in their respective oxygen conditions, in presence of BM-1 containing 100 U/ml heparin. BM-1/heparin media were collected, cells were wash three times with PBS and lysed with RLT buffer (QIAGEN) containing 10 % β-mercaptoethanol.

### RNA isolation and RT-PCR

Total RNA was extracted from cell lysates using QIAGEN RNeasy Mini kits, following the manufacturer’s instructions. Complementary DNA was prepared from 300 ng of total RNA using QIAGEN reverse transcriptase kit, following elimination of genomic DNA using QIAGEN gDNA WipeOut. Since there is no discrepancy between protein level and mRNA expression of Angiopoietin-like 4 (ANGPTL4), only the gene expression was determined [9]. Gene expression was determined by real-time PCR using Eva Green Master Mix (Montreal Biotech) on a Rotor-Gene. Quantitect primers (forward and reverse) for ANGPTL4, metallothionein-3 (MT3), and β-actin were purchased from QIAGEN, with β-actin serving as the reference gene. Delta-delta CT (cycle threshold) analyses were conducted using the Rotor-Gene 6000 software version 1.7.

### LPL activity

LPL activity in differentiated preadipocytes was measured in 50 μl of BM-1-Heparin using the EnzChek Lipase Substrate (Thermo Fisher Scientific), a fluorescent triacylglycerol analog, at a final concentration of 0.62 μM in presence of 18-carbon zwittergent (0.0125 %), 0.15 M NaCl and 20 mM Tris–HCl pH 8. Fluorescence emission kinetics were followed over 1 h at 37 °C and fluorescence from blank wells was subtracted. Average blank-adjusted RFU (relative fluorescence units) are reported here. All samples from an identical experiment were assessed simultaneously, alongside positive controls containing bovine LPL. LPL activity in adipose tissue biopsies was determined similarly, excepted that LPL was first extracted from thawed subcutaneous abdominal adipose tissue samples by incubation at 28 °C for 40 min in Krebs–Ringer buffer containing 1 % BSA (bovine serum albumin) and 0.05 mg/ml heparin as previously described [13, 14].

### In vivo experiments

#### Subjects

Ten healthy young men were recruited from the University of Ottawa population. Study subjects provided written consent and the study protocol was approved by the Research and Ethics Board of the University of Ottawa. Exclusion criteria included: history of physician-diagnosed asthma or other respiratory illness, hypertension, CVD, diabetes, habitual sleep duration of less than 7 h per night, habitual bed time occurring after midnight, shift work, and current smoking habit.

#### Anthropometric measurements

Body weight was determined with a standard beam scale (HR-100, BWB-800AS; Tanita, Arlington Heights, IL) and height was measured using a standard stadiometer (Perspective Enterprises, Portage, Michigan, USA). Waist circumference was measured following World Health Organization procedure. Percentage of fat mass (%FM), total fat mass (FM) and fat free mass (FFM) were measured using dual energy X-ray absorptiometry (DXA) (General Electric Lunar Prodigy, Madison, Wisconsin; software version 6.10.019). Resting energy expenditure (REE) was measured by indirect calorimetry using a Vmax Encore 29 System metabolic cart (VIASYS Healthcare Inc, Yorba Linda, CA).

### Experimental protocol

This was a randomized crossover study consisting of two experimental sessions. Prior to each experimental session, volunteers were counseled to sleep at least 7 h per night, to restrain from any exercises and caffeine for at least 24 h, and to consume a provided standardized evening dinner between 7:00 and 8:00 PM (lasagna of 3220 kJ or 770 kcal; 42 % from carbohydrates, 28 % from fat, and 30 % from protein). On study days, volunteers presented themselves at the laboratory at 7:30 AM after a 12-h overnight fast. Weight measurements were performed before an intravenous line was inserted in the antecubital vein for blood sampling and kept patent with a continuous infusion of 0.9 % saline. A baseline subcutaneous abdominal adipose tissue biopsy (detailed below) was then performed. Volunteers were thereafter asked to consume a fat-rich liquid meal (59 % of calories from fat, 28 % from carbohydrates and 13 % from protein) providing one-third of their estimated daily energy expenditure (obtained by indirect calorimetry during a preliminary session) times a physical activity factor of 1.375 [15], and were then exposed to either intermittent hypoxia or to ambient air (normoxia) for 6 h. Volunteers remained in a semirecumbent position, and occupied themselves by watching television. Sleep was not allowed. Oxyhemoglobin saturation and heart rate were continuously monitored by pulsed oximetry. A second adipose tissue biopsy was performed 3 h after meal ingestion.

### OSA simulation (intermittent hypoxia)

Subjects had to wear a well-fitted oro-nasal mask with a two-way Hans Rudolph non-rebreathing valve connected to an inspiratory line, as reported by Louis et al. [16]. During normoxia session, ambient air only was provided. During intermittent hypoxia sessions, pressurized medical N_2_ was intermittently introduced in the inspiratory line. Oxyhemoglobin saturation (SpO_2_) was allowed to drop to 85 %, at which point the flow of N_2_ was stopped until the oxyhemoglobin saturation returned to the pre-exposure values (~98 %). Intermittent hypoxia was well-tolerated and presented no adverse effects. This experimental setup allowed us to produce 17.3 ± 3.8 hypoxic events per hour, which is comparable to moderate OSA.

### Fasting and postprandial plasma metabolic parameters

Plasma was obtained by centrifugation at 3000 rpm for 10 min at 4 °C immediately after blood collection. Commercially available colorimetric enzymatic assays were used to measure plasma total triglyceride, glucose, non-esterified fatty acid (NEFA) (Wako Chemicals USA Inc, VA, USA) and lactate concentrations (Eton Bioscience Inc. NL, USA). Commercially available enzyme-linked immunosorbent assay kits were used to determine insulin (EMD Millipore, MA, USA) and catecholamines (Rocky Mountain Diagnostics Inc, CO, USA), as previously described [17].

### Subcutaneous abdominal adipose tissue biopsy

On both experimental sessions, two subcutaneous abdominal fat biopsies were performed, one before and one 3 h after meal ingestion. Biopsies were performed in the periumbilical region (within 4–6 cm), as previously described [13]. On the second experimental session, biopsies were performed 4 cm underneath the incisions made on the first session.

### Adipocyte lipolysis

Immediately after the biopsy, roughly 100 mg of fresh adipose tissue, free of capillaries, were digested with collagenase (1 mg/ml) in 4 % BSA Krebs–Ringer buffer at 37 °C and filtered through a nylon mesh. Adipocytes were isolated by centrifugation (500 rpm for 2 min), and washed twice with BSA-Krebs–Ringer buffer. Adipocyte density was then adjusted to 500 adipocytes/50 μl. With constant stirring, 50 μl aliquots of adipocytes suspension were distributed in 1.5 ml Eppendorf tubes and incubated at 37 °C for 2 h in BSA-Krebs–Ringer buffer under 95 % O_2_ in presence of isoproterenol (0.001, 0.01, 0.1, 1 and 10 μM), epinephrine (0.001, 0.01, 0.1, 1 and 10 μM) and UK 14304 (0.0001, 0.001, 0.01, 0.1 and 1 μM). Epinephrine and UK 14304 tubes also contained adenosine deaminase (ADA). Lipolytic rate was determined by glycerol quantification using bioluminescence, as described by Mauriege et al. [18]. Adipocyte density (cells/50 μl) was determined by counting and averaging the number of adipocytes in five 50 μl samples collected throughout the distribution step. Results are presented as μmol of glycerol released by 1 × 10^6^ adipocytes over 2 h. Adipocyte size was obtained by analysing 10× digital images of adipocytes loaded on a hemocytometer using the Infinity Capture and Analyse software (Lumenera Corporation, ON, Canada). Each average adipocyte diameter was computed from at least 150 random individual measurements.

### Statistical analysis

SPSS version 12 for windows was used for data analysis (SPSS Inc. Chicago, IL, USA). Repeated measures analyses of variance (ANOVA) were performed with condition and time as within subject’s parameters. Alpha was set at 0.05.

## Results

### LPL Activity in differentiated human preadipocytes

**Fig. 1:**
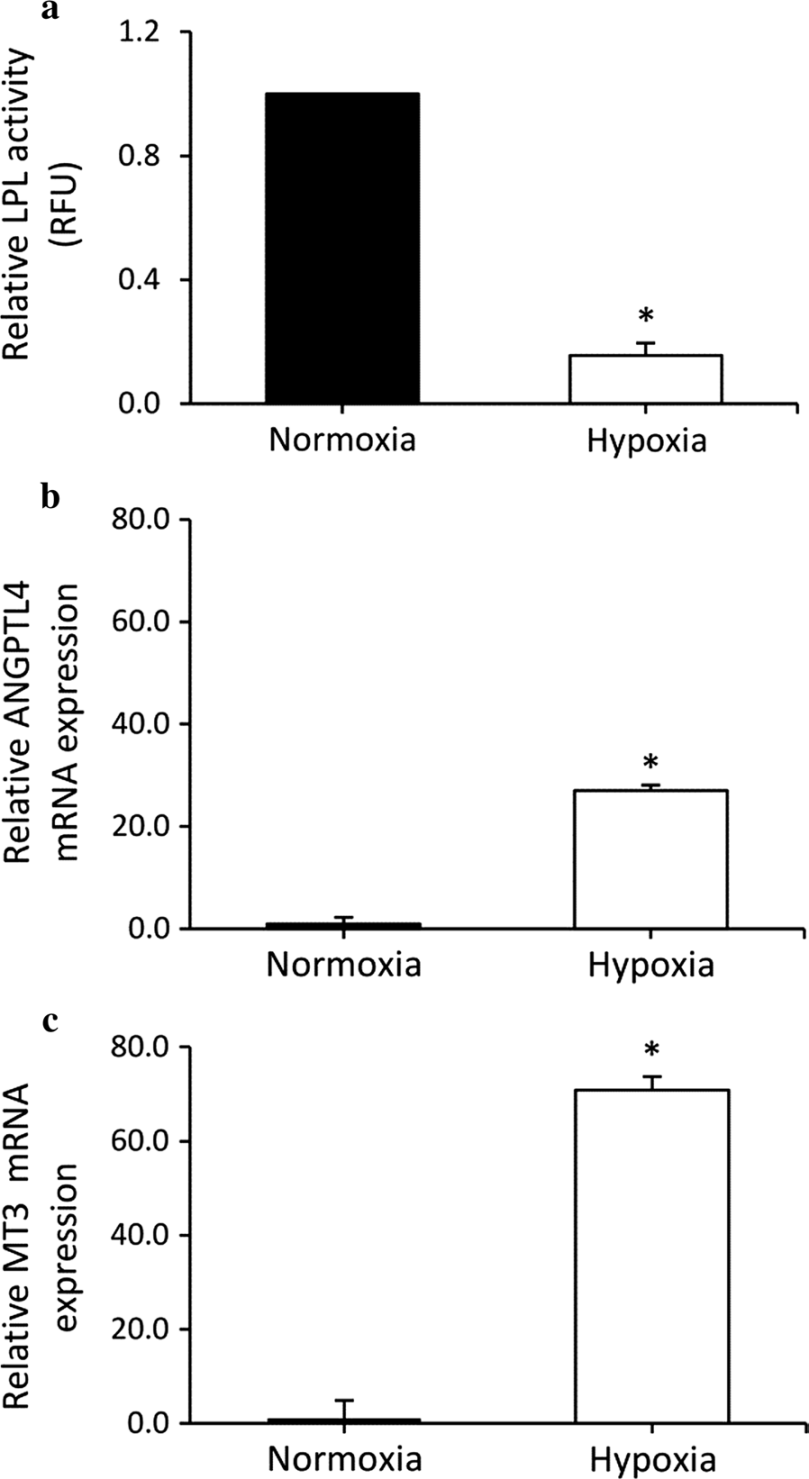
Effect of normoxia (21 % oxygen) or hypoxia (3 % oxygen) on **a** lipopoprotein lipase activity, **b** Angiopoietin like 4 (ANGPTL4) gene expression and **c** metallothionein-3 (MT3) gene expression in differentiated human preadipocytes. Results are from 3 independent experiments performed in triplicate. Values are mean ± standard deviation. Significant difference between experimental sessions at *p < 0.001

In vitro, hypoxia induced a significant 6-fold reduction (p < 0.001) in LPL activity (Fig. 1a). mRNA levels of ANGPTL4, a repressor of LPL activity, and MT3, a gene known to be highly induced by hypoxia, were increased by 27-fold (p < 0.001) and 70-fold (p < 0.001) respectively following hypoxia (Fig. 1b, c).

### Subject characteristics

Metabolic and anthropometric characteristics of the 10 healthy men are represented in Table 1. Participants reported a good quality of sleep, according to the Pittsburgh Sleep Index (3.83 ± 2.71) [19]. On average, participants reported 7.3 h of sleep during the night prior to the experimental sessions. The average time between each experimental session was 7.4 days, and participants’ weight (± 0.35 kg) did not differ between experimental sessions.

**Table 1:** Characteristics of the participants (n = 10 men)

Variable	Mean ± standard deviation
Age (y)	22.8 ± 2.8
Body weight (kg)	84.5 ± 9.8
Height (cm)	181.7 ± 4.7
Body mass index (kg/m^2^)	25.6 ± 2.3
Waist circumference (cm)	84.9 ± 5.1
Fat mass (kg)	12.5 ± 4.5
Lean mass (kg)	69.4 ± 11.2
Body fat (%)	15.3 ± 4.1
Subcutaneous abdominal adipocyte diameter (µm)	72.8 ± 5.7

### Oxyhemoglobin saturation and heart rate responses to intermittent hypoxia

Table 2 displays the variations in heart rate and oxyhemoglobin saturation during normoxia and intermittent hypoxia sessions. During intermittent hypoxia, an average of 17.3 ± 3.8 hypoxic cycles was induced per hour. Heart rate was significantly increased during hypoxic exposure, reaching an average peak increase of ~20 bpm.

**Table 2:** Summary of heart rate and oxyhemoglobin saturation (SpO_2_) during normoxia and intermittent hypoxia sessions

		Normoxia	Intermittent hypoxia
	Exposure time (min)	360.0	350.5 ± 16.7
	Frequency/hour	0	17.3 ± 3.8
	Mean	67.8 ± 11.9	71.7 ± 11.6
Heart rate (BPM)	Maximum	116.0 ± 16.6	120.5 ± 9.2*
	Mean	96.8 ± 1.3	90.2 ± 1.1*
SpO_2_ (%)	Maximum	98.1 ± 0.4	98.4 ± 0.5
	Minimum	93.2 ± 3.9	64.3 ± 5.9*
	≤90 %	0	124.1 ± 31.6
Time SpO_2_ (minutes)	≤85 %	0	50.8 ± 14.5
	≤80 %	0	25.8 ± 7.9

Datas are mean ± standard deviation

* Statistical difference between normoxia and intermittent hypoxia (p < 0.05)

### Plasma metabolic parameters

**Fig. 2:**
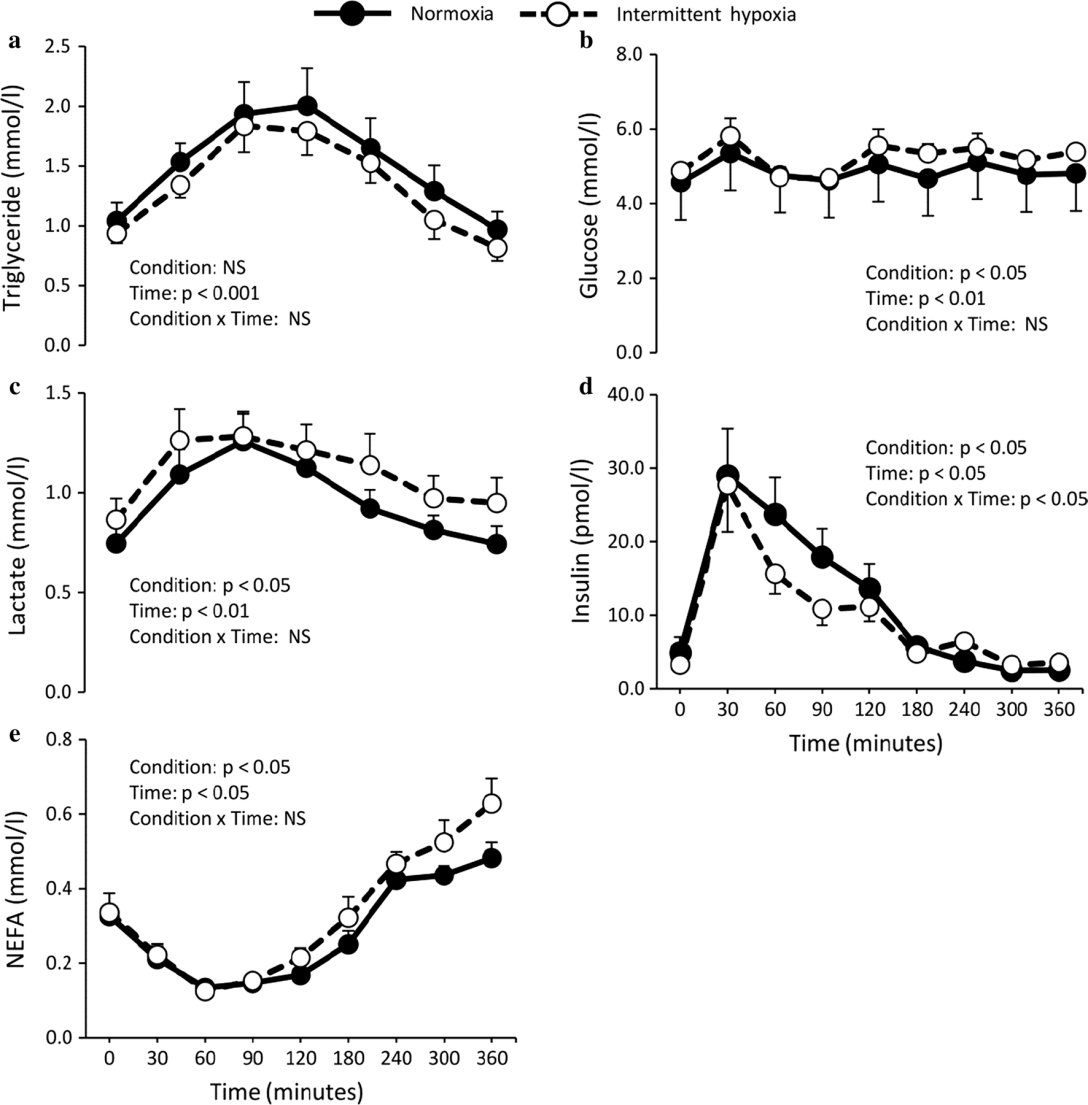
Effect of normoxia or intermittent hypoxia on fasting and postprandial plasma **a** triglyceride, **b** glucose, **c** lactate, **d** insulin and **e** non-esterified fatty acids (NEFA) levels in healthy men. Values are mean ± standard error. *NS* not significant

Postprandial plasma TG, glucose, lactate, insulin, and NEFA levels during normoxia and intermittent hypoxia sessions are depicted in Fig. 2. Postprandially, TG levels increased significantly (time effect, p < 0.001) but did not differ between normoxia and intermittent hypoxia sessions (Fig. 2a). Regardless of time, glucose and lactate were significantly greater during intermittent hypoxia than normoxia (condition effect, p < 0.05). Both variables evolved in a similar manner over time (time effect, p < 0.01) (Fig. 2b, c).

After a peak at 30 min, insulin levels declined more steeply during intermittent hypoxia sessions (condition × time interaction, p < 0.05) (Fig. 2d). Regardless of time, NEFA levels were significantly higher during intermittent hypoxia sessions (condition effect, p < 0.05) (Fig. 2e). No difference in circulating epinephrine and norepinephrine concentrations were observed between experimental conditions (data not shown).

### Subcutaneous adipose tissue metabolism

**Fig. 3:**
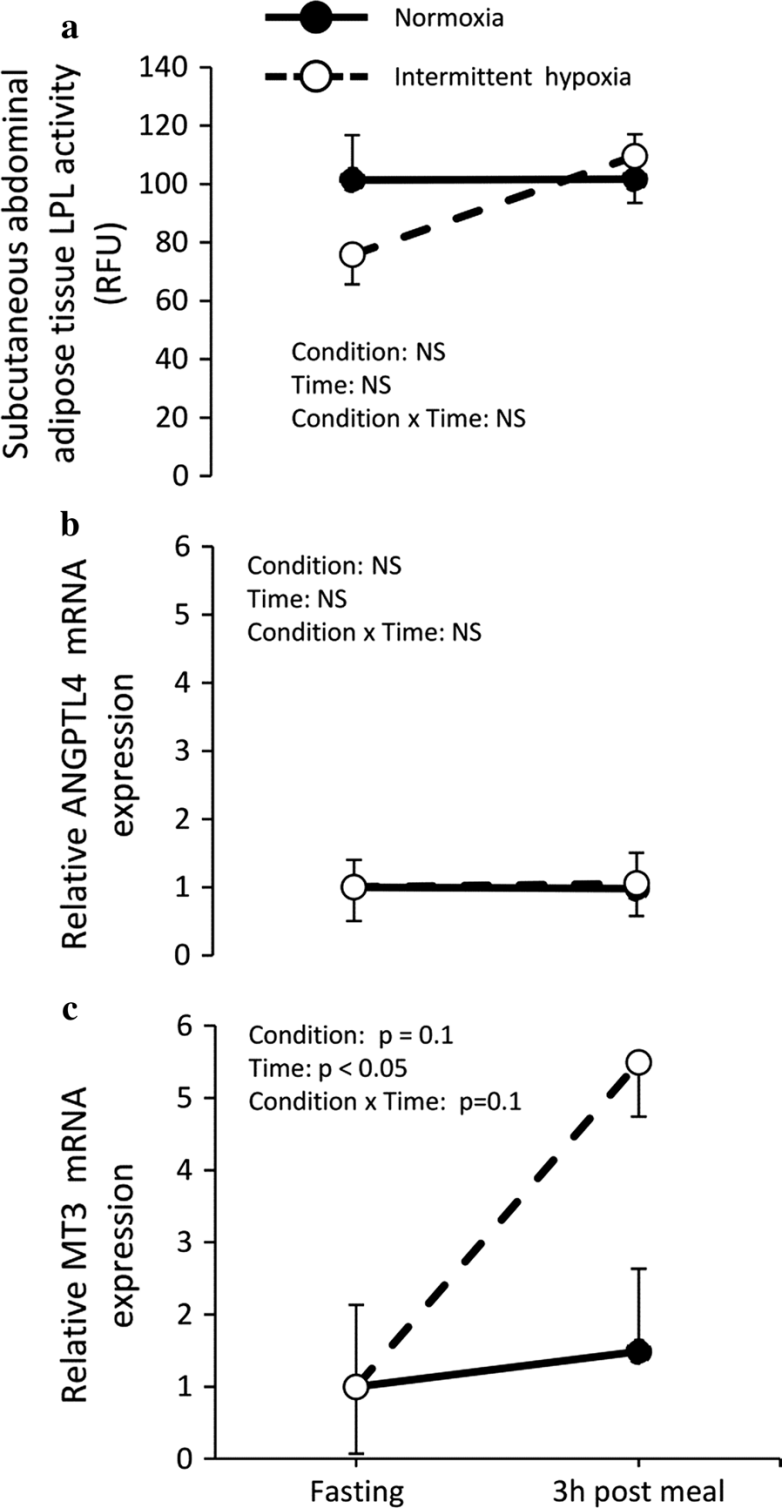
Subcutaneous adipose tissue **a** lipoprotein lipase (LPL) activity, **b** angiopoietin-like 4 (ANGPTL4) gene expression and **c** metallothionein-3 (MT3) gene expression measured before (fasting) and 3 h post meal under normoxia and intermittent hypoxia in healthy men. Values are mean ± standard error. *NS* not significant

Adipose tissue LPL activity (Fig. 3a) and ANGPTL4 expression (Fig. 3b) were affected neither by the meal nor the experimental conditions. Adipose tissue MT3 gene expression levels remain comparable before and after the meal in normoxia, but increased 4-fold under intermittent hypoxia. This interaction fell short of statistical significance (condition × time interaction, p = 0.1) (Fig. 3c).

**Fig. 4:**
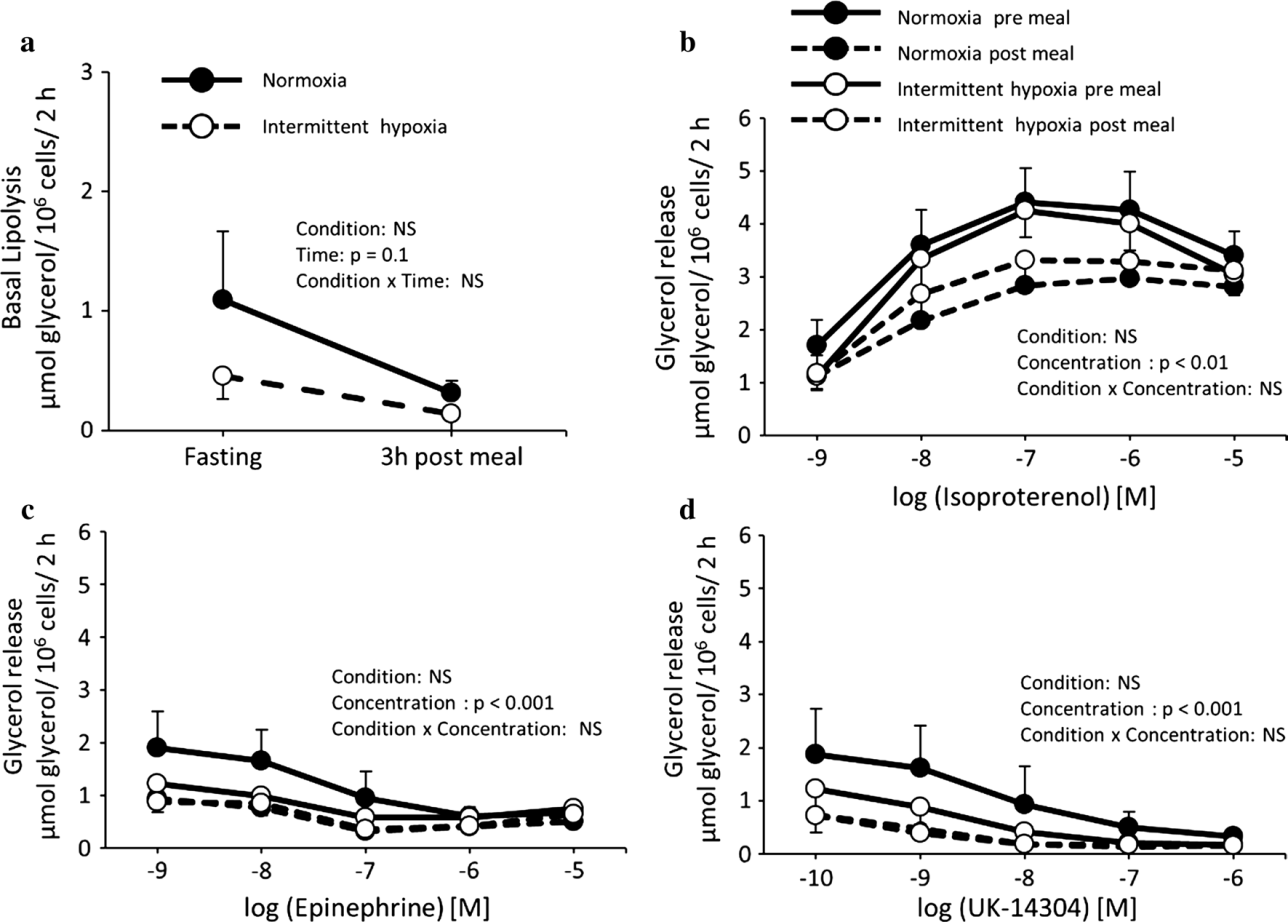
**a** Basal lipolytic rate as well as effect of **b** isoproterenol (β-adrenoreceptor (AR) agonist), **c** epinephrine (mixed α2/β-AR agonist) and **d** UK-14304 (α2- AR agonist) on lipolysis in subcutaneous abdominal isolated adipocytes of healthy men before and 3 h after a meal under normoxia and intermittent hypoxia. Values are mean ± standard error. *NS* not significant

Basal and stimulated lipolytic rate assessed from isolated subcutaneous abdominal adipocytes before and 3 h after the meal are presented in Fig. 4. A trend toward lower basal lipolytic rate in the postprandial phase compared to baseline measurements was observed in both conditions (effect of time, p = 0.1, Fig. 4a). Adenosine deaminase (ADA)-stimulated lipolysis was significantly and similarly reduced postprandially compared to baseline in both conditions (effect of time, p < 0.05) (data not shown). The dose-dependent lipolytic responses to isoproterenol (β-adrenoceptor [AR] agonist) were significantly and similarly reduced postprandially in both conditions (effect of concentration, p < 0.01) (Fig. 4b). Neither the meal nor the conditions affected the antilipolytic effects of epinephrine (mixed α2/β-AR agonist) and UK- 14304 (α2- AR agonist) (effect of concentration, p < 0.001) (Fig. 4c, d).

## Discussion

Using differentiated human preadipocytes and subcutaneous abdominal adipose tissue biopsies from healthy individuals, we investigated the effects of acute hypoxia on adipose tissue lipid storage and/or mobilization functions. We show that 24 h of hypoxia significantly inhibits the activity of a key enzyme involved in adipose tissue TG deposition, LPL, in differentiated human preadipocytes. To explore whether the inhibitory effect of hypoxia on adipose tissue functions are noticeable in humans, young, healthy men were exposed for 6 h to acute intermittent hypoxia, an experimental model that has been proposed to study the metabolic effects of OSA. Acute exposure to intermittent hypoxia was sufficient to alter postprandial NEFA levels, as well as glucose and insulin levels, but did not alter circulating triglycerides nor subcutaneous adipose tissue lipid storage and/or mobilization functions.

### Effects of hypoxia on LPL activity in differentiated human preadipocytes

To our knowledge, this is the first study examining the effects of hypoxia on LPL activity in differentiated human subcutaneous abdominal preadipocytes. Our results show a 6-fold reduction in LPL after a 24 h-incubation in hypoxic conditions. Consistently, ANGPTL4, a major post-translational regulator of LPL activity which inactivates LPL at the plasma membrane of adipocytes [20], was significantly increased after hypoxia, as previously reported by Wood et al. [21]. These observations confirm that the potential for lipid uptake of differentiated human preadipocytes is sensitive to an acute decrease in oxygen availability. It also complements recent evidence indicating that hypoxia impedes expression level of genes involved in de novo lipogenesis in human visceral adipose tissue [22].

### Metabolic (non-lipid) effects of intermittent hypoxia in humans

In order to determine whether the reduction in LPL activity, observed in differentiated preadipocytes exposed to hypoxia, is translated in vivo, 10 young, healthy men were exposed to intermittent hypoxia in the postprandial state. Intermittent hypoxia was chosen over chronic hypoxia based on its similarity to sleep apnea, a disorder that is associated with an altered lipid profile [3, 23]. A fat-rich meal was also given to our participants based on numerous animal studies suggesting that postprandial triglyceride clearance is impaired by hypoxia [8, 10]. Our experimental setup clearly induced a systemic response: besides oxyhemoglobin desaturation cycles (by design), heart rate sharply and systematically increased during hypoxic cycles, reflecting a hypoxia-induced increase in sympathetic tone. As compared to values observed in normoxia condition, glucose and lactate levels were significantly increased after 90 min of intermittent hypoxia exposure, likely reflecting a shift in energy substrate utilization. Any changes in energy substrate partitioning, however, were impossible to confirm by indirect calorimetry, due to the constant changes in inspired and expired gas mixture.

### Effects of intermittent hypoxia on lipid and adipose tissue metabolism

No significant difference in postprandial triglyceridemia excursion was observed during intermittent hypoxia. Consistently, postprandial LPL activity, measured from adipose tissue biopsies, was not different between normoxia and intermittent hypoxia conditions. Despite a 4-fold increase in abdominal subcutaneous adipose tissue MT3 expression, which likely suggests that adipose tissue have been exposed to reduced partial pressure in oxygen, ANGPTL4 expression was not induced by the intermittent hypoxia session. The absence of changes in LPL activity and ANGPTL4 expression suggests that the clearance rate of TG by adipose tissue was likely not affected by intermittent hypoxia in our study sample. These results are not consistent with those from animal studies (mice) demonstrating that acute exposure to hypoxia [10] or chronic intermittent hypoxia [8, 9] delays plasma TG clearance and decrease subcutaneous LPL activity in white adipose tissue following a meal. These discrepancies, if not species-related, may be explained by the severity of the hypoxic stress. While the current study was conducted with intermittent hypoxia at a rate of 17.3 ± 3.8 events/hour for 6 h, Drager et al. [9] conducted their animal studies with a frequency of 60 hypoxic events/hour and Jun et al. [10] used constant hypoxia for 6 h.

The slight but statistically significant increase in plasma NEFA after 120 min of intermittent hypoxia is in line with several past observations of increased NEFA in animals exposed to hypoxic conditions [10]. This is typically explained by an increase in sympathetic tone, which stimulates adipose tissue lipolysis [10]. Results of lipolytic responses in isolated adipocytes from adipose tissue biopsies suggest, however, that if an increase in lipolysis rate occurred in vivo, it did not translate into an altered ex vivo response to lipolysis stimulating/inhibiting agents. Instead, it appears that the meal provided to our participants had a clear inhibiting impact on the adipocyte lipolytic activity. To the best of our knowledge, this is the first study to report ex vivo lipolytic response in adipocytes before and after the consumption of a meal. Our observations clearly support a strong suppression of NEFA release by isolated adipocyte of lean individuals in the postprandial phase. It is important to note, however, that despite the clear postprandial inhibition of lipolysis, adipocytes were still responsive to epinephrine and isoproterenol. Accordingly, the elevated plasma NEFA levels observed during intermittent hypoxia could still come from an increase in sympathetic drive, which should have been less present in the normoxia session. Other contributing factors to the increase in plasma NEFA during the intermittent hypoxia session include an earlier relief of lipolysis inhibition by insulin, and/or a decrease in circulating fatty acid utilisation by peripheral organs, leading to their accumulation in circulation. An increase in NEFA levels, in the long term, could lead to an increase in concentration of very low-density lipoprotein, small dense low-density lipoprotein particles, and elevated apolipoprotein B concentrations in plasma, all of which are associated with increased risk of coronary heart disease and stroke [24].

Some limitations of this study warrant discussion. First, in our in vitro experiments, only two different oxygen concentrations were tested: 3 % and 21 % O_2_. Since it has been reported that adipocytes are sensitive to even relatively small changes in oxygen level within the physiological range [12, 25], further studies with different concentrations of oxygen could be undertaken. Limitations of our in vivo studies includes: the duration of intermittent hypoxia, which was brief and limited to only 6 h in order to limit burden and potential side-effects on the hypoxia-naïve participants; the severity of the intermittent hypoxia, which was equivalent to moderate OSA; and the homogeneity of our study sample, which consisted exclusively of healthy young men [2]. All these limitations limit the generalization of our metabolic observation to individuals suffering from OSA. OSA patients are likely exposed to intermittent hypoxia on a daily basis, and a large proportion of them exhibit metabolic complications [26]—increased adiposity, dyslipidemia, and insulin resistance (consequently of OSA or not)—that may synergistically exacerbate the negative lipid-altering effects of intermittent hypoxia. Finally, adipose tissue LPL activity is both sex and depot sensitive [14]. One could argue that these confounding factors may explain part of the discrepancy between our in vitro and in vivo observations since preadipocytes were obtained from female donors, while our in vivo experiments included only male subjects. While it is possible that sex and depot can affect adipocytes responses to hypoxia, it should be emphasized that our in vitro approach served only as a proof of concept that differentiated human fat cells, regardless of the donor's sex or adipose tissue depot, show a reduction in LPL activity under hypoxia. Regarding our choice of sampling site, the periumbilical region was chosen because the subcutaneous abdominal adipose tissue is responsible for most (45–50 %) of the clearance of exogenous lipids in humans [27, 28]. The remaining of the postprandial triglyceride clearance is proposed to be LPL-mediated in various other sites such as the subcutaneous femoral and visceral adipose tissues as well as the heart. Future studies remain to be performed to investigate how these other sources of LPL activity could be affected by intermittent hypoxia and to examine whether intermittent hypoxia affects the various sources of LPL similarly in men and women.

## Conclusions

Our in vitro results indicate that hypoxia significantly inhibits lipoprotein lipase activity in differentiated human preadipocytes, while in vivo observations show that an acute session of intermittent hypoxia significantly increases postprandial NEFA levels, but not postprandial circulating TG, adipose tissue LPL activity, or adipocyte lipolysis, in healthy young men.
