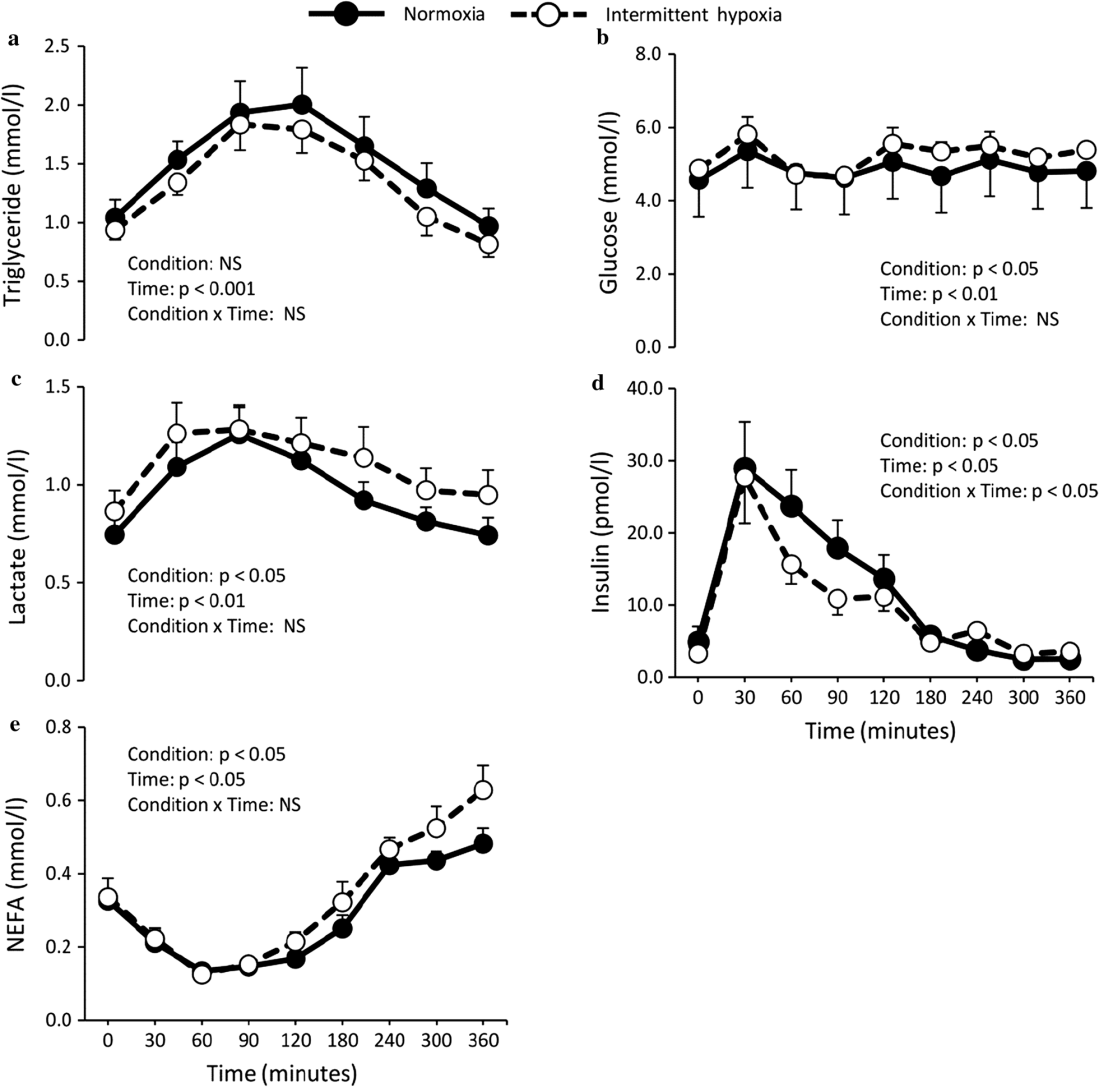# Correction to: Effects of acute hypoxia on human adipose tissue lipoprotein lipase activity and lipolysis

**DOI:** 10.1186/s12967-021-02758-w

**Published:** 2021-04-01

**Authors:** Bimit Mahat, Étienne Chassé, Jean-François Mauger, Pascal Imbeault

**Affiliations:** Behavioral and Metabolic Research Unit, School of Human Kinetics, Faculty of Health, Sciences, University of Ottawa, 125, University Street (room 350), Ottawa, ON K1N 6N5 Canada

## Correction to: J Transl Med (2016) 14:212 10.1186/s12967-016-0965-y

Following publication of the original article [1], the authors identified an error in Fig. 2. In Fig. 2d, the units for insulin should be uU/ml, not pmol/l as originally stated. The incorrect and correct figure are included in this Correction article. The original article has been updated.

**Correct Figure 2:**

**Fig. 2:**
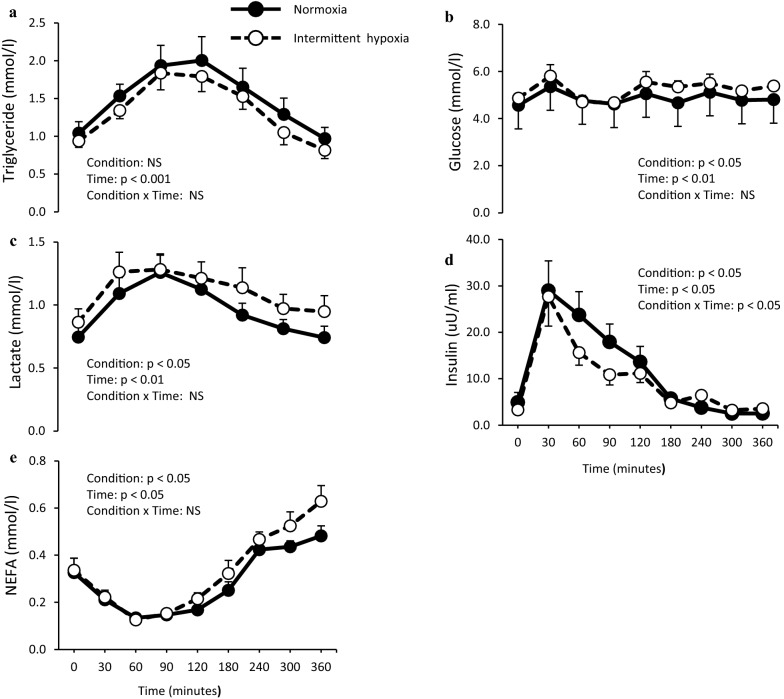
Effect of normoxia or intermittent hypoxia on fasting and postprandial plasma **a** triglyceride, **b** glucose, **c** lactate, **d** insulin and **e** non-esterified fatty acids (NEFA) levels in healthy men. Values are mean ± standard error. *NS* not significant

**Incorrect Figure 2:**